# Two Extracellular Poly(*ε*-caprolactone)-Degrading Enzymes From *Pseudomonas hydrolytica* sp. DSWY01^T^: Purification, Characterization, and Gene Analysis

**DOI:** 10.3389/fbioe.2022.835847

**Published:** 2022-03-18

**Authors:** Linying Li, Xiumei Lin, Jianfeng Bao, Hongmei Xia, Fan Li

**Affiliations:** ^1^ School of Life Sciences, Northeast Normal University, Changchun, China; ^2^ Engineering Research Center of Glycoconjugates, Ministry of Education, Changchun, China; ^3^ Changchun GeneScience Pharmaceutical Co., Ltd., Changchun, China; ^4^ National Demonstration Center for Experimental Biology Education, Northeast Normal University, Changchun, China

**Keywords:** poly(*ε*-caprolactone), *Pseudomonas hydrolytica*, PCL-degrading enzyme, cutinase, lipase

## Abstract

Poly(ε-caprolactone) (PCL) is an artificial polyester with commercially promising application. In this study, two novel PCL-degrading enzymes named PCLase I and PCLase II were purified to homogeneity from the culture supernatant of an effective polyester-degrading bacterium, *Pseudomonas hydrolytica* sp. DSWY01^T^. The molecular masses of PCLase I and PCLase II were determined to be 27.5 and 30.0 kDa, respectively. The optimum temperatures for the enzyme activities were 50 and 40°C, and the optimum pH values were 9.0 and 10.0, respectively. The two enzymes exhibited different physical and chemical properties, but both enzymes could degrade PCL substrates into monomers and oligomers. Weight loss detection and scanning electron microscopy revealed that PCLase I had more effective degradation ability than PCLase II. The genes of the two enzymes were cloned on the basis of the peptide fingerprint analysis results. The sequence analysis and substrate specificity analysis results showed that PCLase I and PCLase II were cutinase and lipase, respectively. Interface activation experiment also confirmed this conclusion. Structural analysis and modeling were further performed to obtain possible insights on the mechanism.

## Introduction

In recent years, biodegradable and biocompatible polyesters such as poly(3-hydroxybutyrate) (PHB), poly(butylene succinate) (PBS), poly(*ε*-caprolactone) (PCL), and poly(lactic acid) (PLA) have been widely used in packaging, medical, and ecological applications due to the adverse effects of traditional plastics on the environment (Ikada and Tsuji, 2000; Gross and Kalra, 2002; Woodruff and Hutmacher, 2010; Gautam et al., 2013). Among these materials, PCL is a semi-crystalline linear aliphatic polyester that is ring-opening polymerized by *ε*-caprolactone (Lovera et al., 2007). The excellent properties of PCL, including its biocompatibility, low melting point, and high processibility, make it useful in different industries.

PCL can be completely degraded by microorganisms in the environment, but the speed of its degradation varies due to environmental differences (Min et al., 2007; Cho et al., 2011). Various PCL-degrading bacteria or fungi have been isolated, and previous research have shown that PCL-degrading enzymes from microorganisms mediate PCL degradation (Murphy et al., 1996; Murphy et al., 1998; Khatiwala et al., 2008; Sekiguchi et al., 2011; Abdel-Motaal et al., 2014). Previous works have reported that some PCL-degrading enzymes are lipases, whereas some are cutinases that degrade cutin under natural conditions. However, existing research still focuses mainly on the evaluation of PCL degradation performance, and the systematic investigation of PCL-degrading enzymes’ mechanism is limited.

The biodegradation of PCL is an unusual feature considering that PCL is a chemically synthesized polymer. PCL’s hydrolysis mechanism by various enzymes can provide valuable information for the synthesis of novel biodegradable materials. PCL-degrading enzymes derived from microorganisms are also environment-friendly catalysts that can be used in polyester recycling, and these enzymes can be effectively applied to the controlled degradation and recycling of PCL. Several studies have recently been conducted on the use of PCL degradation in blended materials for the preparation of porous scaffolds or modification of polymers (Kikkawa et al., 2010; Ju et al., 2014). The exploitation and application of different types of PCL-degrading enzymes are indispensable in these studies.

In a previous work, we screened a bacterial strain that can degrade several kinds of polyesters from activated sludge. The strain was identified to be a new species of *Pseudomonas* named *Pseudomonas hydrolytica* sp. DSWY01^T^ (Zhou et al., 2020). In the current study, two PCL-degrading enzymes were simultaneously purified from the strain; their enzymatic properties and PCL degradation behavior were examined. The possible mechanism of enzyme function was determined according to the enzyme’s sequence and structural information.

## Materials and Methods

### Chemicals, Materials, and Methods

PCL with a molecular weight of 80,000 g/mol was obtained from Solvay Interox Ltd., and PCL film prepared by hot pressing was provided by Changchun Institute of Applied Chemistry. Plysurf A210G was acquired from Daiichi Kogyo Seiyaku (Japan). DEAE Sepharose Fast Flow and Sephadex G-75 columns were obtained from GE Healthcare Bio-Sciences AB (Sweden). Unless otherwise stated, all chemicals used were of analytical grade.

### Bacterial Strains, Plasmids, and Medium

*P. hydrolytica* DSWY01^T^ was screened and preserved in our laboratory. A mineral medium containing 0.1% PCL, 1.194% Na_2_HPO_4_·12H_2_O, 0.554% KH_2_PO_4_, 0.1% NH_4_Cl, 0.05% MgSO_4_·7H_2_O, and 0.0005% CaCl_2_·2H_2_O was used for the strain cultivation. The gene was cloned into plasmid pET-22b (+) and transformed into competent cells of *Escherichia. coli* BL21 (DE3) for heterologous expression.

### Purification of PCL-Degrading Enzymes

*P. hydrolytica* sp. DSWY01^T^ was fermented in PCL-emulsified medium at 37°C for 48 h, and the culture supernatant was obtained by centrifugation at 12,000 rpm for 20 min. After being dialyzed against 20 mM Na_2_HPO_4_-NaH_2_PO_4_ buffer (pH 7.6), the sample was applied to DEAE Sepharose Fast Flow (1.0 × 20 cm) (pH 7.6) and eluted with a linear gradient of NaCl from 0 to 1.0 M. The unpurified active fractions were further applied to DEAE Sepharose Fast Flow (1.0 × 20 cm) (pH 9.0) or Sephadex G-75, which were eluted with NaCl (0–1.0 M) and 0.02 M phosphate buffer (pH 7.6), respectively. All of the steps were performed at 4°C.

### Enzyme Assay and Protein Measurement

Initially, 0.1% (w/v) of PCL was emulsified with 0.01% (w/v) of Plysurf A210G and used as the substrate. PCL-degrading enzyme at appropriate concentration was mixed with the substrate and kept at 50°C for 20 min to assay the activity. The decrease in the PCL emulsions’ turbidity was measured using a UV spectrophotometer. One unit (U) of enzymatic activity was defined as the amount of enzyme required to reduce absorbance by 0.001 at 650 nm per min (Shirakura et al., 1986). The protein concentration was determined *via* Coomassie Brilliant Blue method, with bovine serum albumin as the standard.

### Effects of pH and Temperature on the PCL-Degrading Activity of the Purified Enzymes

The PCL-degrading activity of the purified enzymes was assayed in 0.2 M buffers of various pHs (citrate buffer, pH 4–6; Na_2_HPO_4_-NaH_2_PO_4_ buffer, pH 6–8; Tris-HCl buffer, pH 8–9; Gly-NaOH buffer, pH 9–12) to determine the optimum pH. The optimal temperature was determined by measuring the enzyme’s activity at temperatures ranging from 30 to 75°C. To evaluate the pH stability and thermostability of the purified enzymes, the enzyme was kept either at a pH ranging from 3 to 12 at 4°C for 24 h or at temperatures ranging from 20 to 70°C for 2 h; residual activity was then assayed under standard conditions.

### Effects of Metal Ions, Inhibitors, and Organic Solvents on the PCL-Degrading Activity of the Purified Enzymes

Several metal ions, including Mg^2+^, Na^+^, Ca^2+^, Zn^2+^, Co^2+^, Fe^3+^, Fe^2+^, Cu^2+^, and Mn^2+^, ethylenediaminetetraacetic acid (EDTA), and PMSF, were added to the reaction with final concentrations of 1 mM or 10 mM to determine their effects on the PCL-degrading activity of the purified enzymes. The effects of various chemical reagents, including methanol, ethanol, glycerol, Tween-80, and Triton X-100, were assayed with final concentrations of 1% (v/v) and 10% (v/v). The activity of the enzyme incubated without any metal ion and chemical was considered 100%.

### Substrate Specificity of the PCL-Degrading Enzymes

PLA, PHB, PBS, tributyrin, olive oil, apple peel cutin, and various *p*NP esters, including *p*NP acetate (C2), *p*NP butyrate (C4), *p*NP caprylate (C8), *p*NP laurate (C12), and *p*NP myristate (C14), were used as substrates to determine the substrate specificity of PCL-degrading enzyme. The degradation of PLA, PHB, PBS, and *p*NP esters was assayed using a spectrophotometer (Kazenwadel et al., 2012). The degradation of tributyrin and olive oil was determined by titration (Prazeres et al., 2006), and the degradation of cutin was determined by weighing method.

### Determination of Hydrolysis Products of the PCL-Degrading Enzymes

PCL-degrading enzymes was incubated with PCL-emulsified substrate at 50°C for 30 min, and the supernatant was collected and analyzed by tandem quadrupole mass spectrometry (MS) (Quattro Premier XE) with capillary voltage of 3.0 kV, cone voltage of 20 V, and source temperature of 110°C. The same sample incubated with enzyme inactivated by boiling was used as a control.

### Degradation of Poly(ε-Caprolactone) Film by the PCL-Degrading Enzymes

PCL films (10 × 10 × 0.2 mm) were incubated with 1 ml of PCL-degrading enzyme (0.2 mg/ml) at 45°C. The enzyme solution was changed every 24 h, and the films were taken out at the same interval to be completely vacuum dried at room temperature. The film’s weight was then measured to obtain the weight loss curve (Shi et al., 2020).

### SEM Observations

The surface morphologies of the PCL films with different degradation degrees were observed through a Hitachi S570 SEM (Japan) at an acceleration voltage of 15 kV. The surface of each PCL film was sprayed with gold before analysis.

### Gene Cloning and Sequence Analysis

The protein band of the PCL-degrading enzyme was excised from SDS-PAGE gel. After trypsin digestion, the sample was subjected to matrix-assisted laser desorption/ionization time-of-flight MS (MALDI-TOF-MS, 4,700 Proteomics Analyzer, Tianjin Biotechnology Inc., China). The results were compared and analyzed in MASCOT Peptide Mass Fingerprint database. Primers were designed on the basis of the sequence with 100% identity in the database, and the PCL-degrading enzyme gene fragments were amplified by PCR using strain genome as template. The domain of the PCL-degrading enzyme was predicted by Inter Pro Scan, and the signal peptide was predicted by Signal P4.1 Server. The secondary structure was predicted on the online software PSIpred, and the three-dimensional spatial structure was found in UniProt protein database or constructed using SWISS-MODEL.

## Results

### Purification of PCL-Degrading Enzyme

**FIGURE 1:**
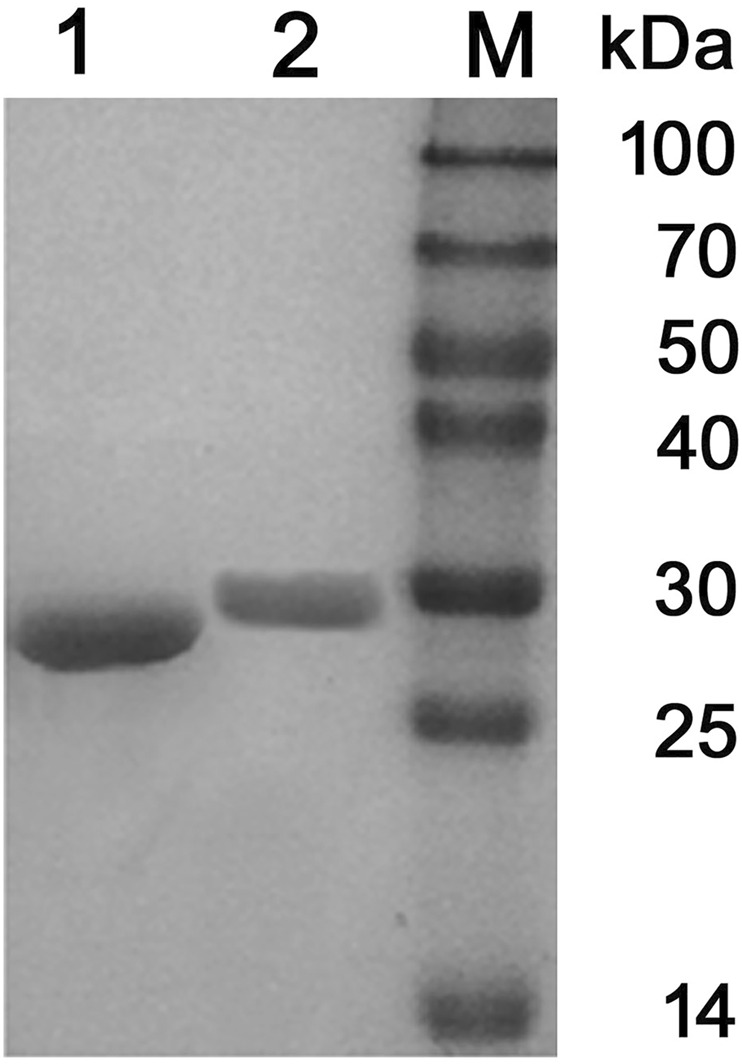
SDS-PAGE analysis of the purified PCL-degrading enzymes. (Lane 1: purified PCLase I; Lane 2: purified PCLase II; Lane M: molecular weight marker).

Two PCL-degrading enzymes were purified to homogeneity from the culture supernatant of *P. hydrolytica* sp. DSWY01^T^ as described in “Materials and methods.” The component named PCLase I was purified by a combination of DEAE-Sepharose chromatographic steps eluted at pH 7.6 and pH 9.0, respectively, and another component named PCLase II was obtained by a combination of DEAE–Sepharoseand further Sephadex G-75 column chromatography. Table 1 summarizes the enzymes’ purification. The molecular masses of PCLase I and PCLase II were determined by SDS-PAGE analysis to be 27.5 and 30.0 kDa, respectively (Figure 1).

**TABLE 1:** Purification of the PCL-degrading enzymes from the supernatant of *pseudomonas hydrolytica* sp. DSWY01^T^.

Components	Steps	Total proteins (mg)	Total activities (U)	Specific activities (U/mg)	Purification (fold)	Yield (%)
Culture supernatant		120.9	19,945.5	164.9	1.0	100.0
lyophilized dialysate		43.9	12880.4	293.4	1.8	64.6
PCLase I	DEAE-Sepharose (pH7.6)	21.8	7,384.7	338.7	2.1	37.0
	DEAE-Sepharose (pH9.0)	4.5	3,728.9	828.6	5.0	18.7
PCLase II	DEAE-Sepharose (pH7.6)	18.0	5,884.7	326.9	2.0	29.5
	Sephadex G-75	3.9	2,578.9	661.3	4.0	12.9

### Effects of Temperature and pH on the PCL-Degrading Activity of Purified Enzymes

**FIGURE 2:**
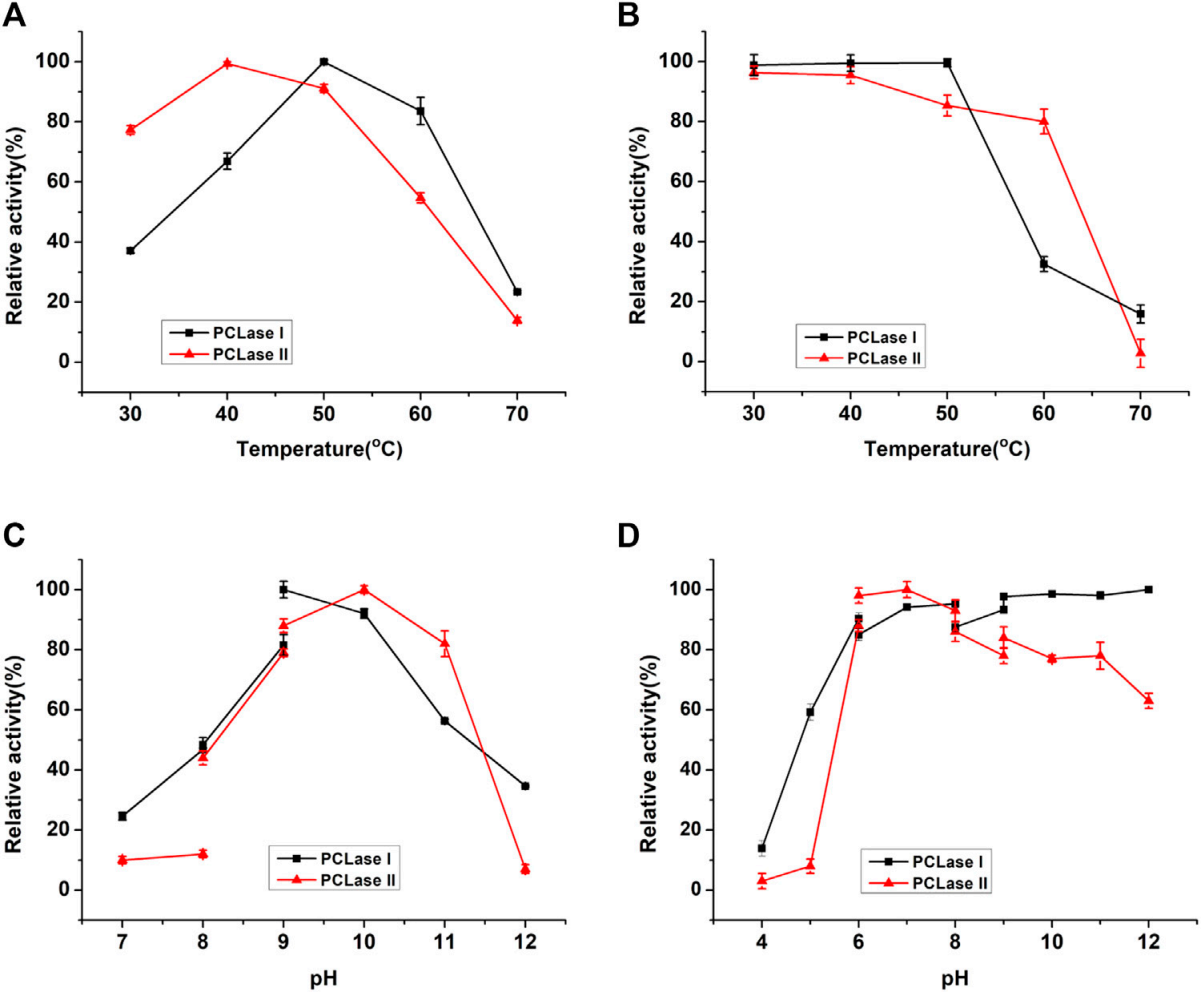
Effects of temperature and pH on the enzymatic activity of the purified PCL-degrading enzymes. **(A)** Temperature dependence of enzymatic activity; **(B)** Thermostability of the purified enzymes; **(C)** pH dependence of enzymatic activity; **(D)** Stability of the purified enzymes at different pH conditions.

The PCL-degrading activities of the purified enzymes were detected at different temperatures ranging from 20 to 70°C. The purified PCLase I and PCLase II exhibited the maximum degrading activity at 50 and 40°C, respectively (Figure 2A). The two PCL-degrading enzymes were moderately stable at temperatures up to 50°C. However, when the temperature increased to 60°C, the activity of PCLase I was almost lost after 2 h of incubation, whereas PCLase II retained more than 80% of the enzymatic activity (Figure 2B).

The optimum pH for PCLase I and PCLase II were observed to be pH 9.0 and pH 10.0, respectively, and almost no activity was detected under acidic conditions (Figure 2C). PCLase I and PCLase II were stable at pH conditions ranging from 6.0 to 12.0, and PCLase I showed remarkable stability at pH 12.0 with activity of approximately 100% (Figure 2D).

### Effects of Metal Ions and Chemicals on the PCL-Degrading Activity of Purified Enzymes

The effects of various metal ions on the activity of the purified enzymes are listed in Table 2. The results showed that 1 mM of Mg^2+^ and Ca^2+^ significantly enhanced the activity of the two PCL-degrading enzymes. When the ion concentration was increased to 10mM, these two ions had a stronger promotion on PCLase I, reaching 833 and 226% of the original activity. However, these ions significantly inhibited the activity of PCLase II with approximately 60% activity remaining. In addition, 1 mM of Fe^3+^ and Fe^2+^ significantly enhanced PCLase I’s activity while inhibiting PCLase II’s activity to be 80.77 and 91.16%, respectively. By contrast, Co^2+^ and Cu^2+^ enhanced the activity of PCLase II and inhibited that of PCLase I. Specifically, Co^2+^ completely inactivated PCLase I. These difference indicate the amino acids involved in the degradation function of the two enzymes may be different in property.

**TABLE 2:** Effects of metal ions on the activity of the two PCL-degrading enzymes.

Metal ions	Residual activity (%)			
	PCLase I		PCLase II	
	1 mM	10 mM	1 mM	10 mM
Mg^2+^	169.80 ± 4.90	833.60 ± 3.20	220.57 ± 0.90	61.06 ± 2.39
Na^+^	106.60 ± 0.60	88.00 ± 1.20	115.50 ± 0.49	64.98 ± 2.21
Ca^2+^	149.00 ± 1.20	226.30 ± 2.30	132.55 ± 1.64	66.80 ± 2.73
Zn^2+^	17.30 ± 1.00	-	22.48 ± 0.57	-
Co^2+^	0	-	110.38 ± 0.71	-
Fe^3+^	227.80 ± 16.50	-	80.77 ± 0.99	-
Fe^2+^	166.00 ± 1.90	-	91.16 ± 0.54	-
Cu^2+^	88.20 ± 0.50	-	108.99 ± 0.36	-
Mn^2+^	41.50 ± 2.40	-	99.37 ± 1.18	-

- Means that the effect of the metal ion could not be detected for the precipitate.

Table 3 shows the effects of chemicals on the purified enzymes. PCLase I and PCLase II were tolerant to tested organic solvents and had more than 80% activity remaining at 1 and 10% concentrations, respectively. Surfactant Triton X-100 strongly inhibited the activity of these two PCL-degrading enzymes, and almost no activity was detected even at 1% concentration. Tween-80 at 1% concentration slightly inhibited the activity of PCLase I, but almost completely inhibited that of PCLase II; when the concentration was increased to 10%, the inhibition on both enzymes was significant. These results indicated that the active centers of the two PCL-degrading enzymes may have a hydrophobic region that is necessary for their functions.

**TABLE 3:** Effects of chemicals on the activity of the two PCL-degrading enzymes.

Organic solvent	Residual activity (%)			
	PCLase I		PCLase II	
	1%	10%	1%	10%
Methanol	84.50 ± 0.49	128.10 ± 1.50	95.34 ± 0.60	87.06 ± 1.35
Ethanol	96.60 ± 0.80	125.50 ± 3.30	91.71 ± 0.56	83.42 ± 0.60
Glycerol	108.80 ± 1.30	108.10 ± 1.40	95.19 ± 1.27	81.95 ± 1.43
Tween-80	86.80 ± 0.20	0	1.85 ± 0.72	1.78 ± 1.93
Triton X-100	0	0	0.98 ± 0.15	0.63 ± 0.45

### Substrate Specificity of the Purified Enzymes

The activities of PCLase I and PCLase II were tested on several representative substrates. Table 4 shows that aside from PCL, these two PCL-degrading enzymes could also degrade PBS, *p*NP ester, tributyrin, and olive oil, while neither enzyme degraded PLA. In addition, PCLase I degraded crude cutin and did not degrade PHB; by contrast, PCLase II degraded PHB while had no degrading activity on cutin. These results indicated that PCLase I may be a cutinase, whereas PCLase II is an esterase that does not degrade cutin.

**TABLE 4:** Substrate specificity of the two PCL-degrading enzymes.

Substrate	PCLase I	PCLase II
PCL	+	+
PHB	−	+
PLA	−	−
PBS	+	+
*p*NPC2-C16	+	+
Tributyrin	+	+
Olive oil	+	+
Crude cutin	+	−

+ Means that the enzyme has the degrading ability, and − means no degrading ability.

### Determination of Enzymatic Hydrolysis Products

**FIGURE 3:**
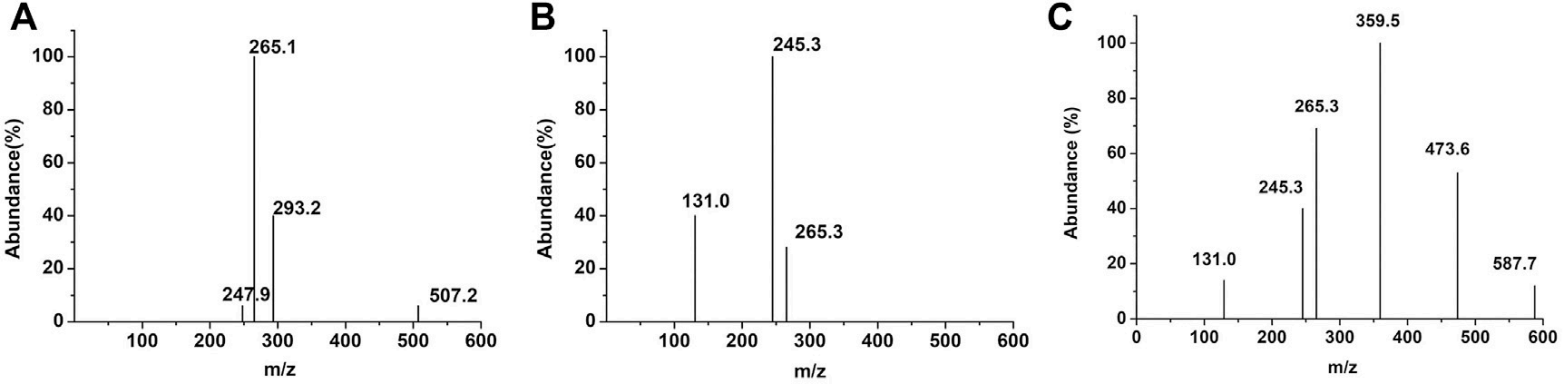
MS results of the degradation products of PCL-degrading enzymes. [**(A)**: control group; **(B)**: PCLase I; **(C)**: PCLase II].

The products of PCL degradation by purified PCL-degrading enzymes were identified by MS. Figure 3 shows that unlike the control group, monomers (peak at m/z 131.0), dimers (peak at m/z 245.3), trimers (peak at m/z 359.5), and tetramers (peak at m/z 473.6) were detected in the samples. These results indicated that the catalytically active centers of the two purified PCL-degrading enzymes may be able to bind to long-chain substrates containing several PCL monomers and cleave the ester bonds in them. This mode is different with several reported PHB depolymerases which can only cleave the substrate from the chain end and release monomers or dimers (Zhou et al., 2009; Wang et al., 2012; Li et al., 2018).

### Degradation and SEM Observation of the PCL Films Degraded by Purified Enzymes

**FIGURE 4:**
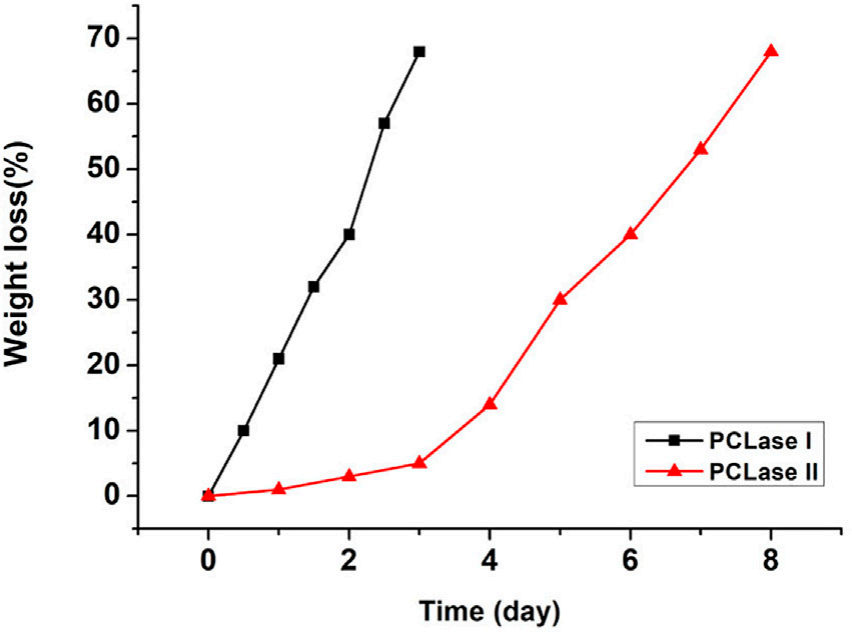
Weight loss of the degraded PCL films.

The weight loss of the films caused by enzymatic hydrolysis can be used as an index to measure the enzymatic degradation ability. Figure 4 presents the weight loss plot of the PCL films with degradation time. The relationship between the weight loss of the PCL films and degradation time was close to linear for PCLase I, and the loss of the films degraded by PCLase I reached 70% after 3 days of incubation. This degradation trend is consistent with the result of cutinase degradation of the PCL film reported by Shi (Shi et al., 2020). However, the degradation profile of PCLase II is different from that of PCLase I. Its plot could be divided into a slow stage (0–3 days) and a fast stage (3–8 days) (Figure 4). The weight loss was achieved at approximately 75% after 8 days of incubation. These results showed that different enzymes have varying degradation rates for PCL solid materials, which may be attributed to different enzymatic hydrolysis modes. Shi also has similar findings, but the difference from the results of the current study is that the speed of PCL degradation by *Candida antarctica* lipase that Shi used was initially fast and then slowed down (Shi et al., 2020); additionally, the specific mechanism requires further research.

**FIGURE 5:**
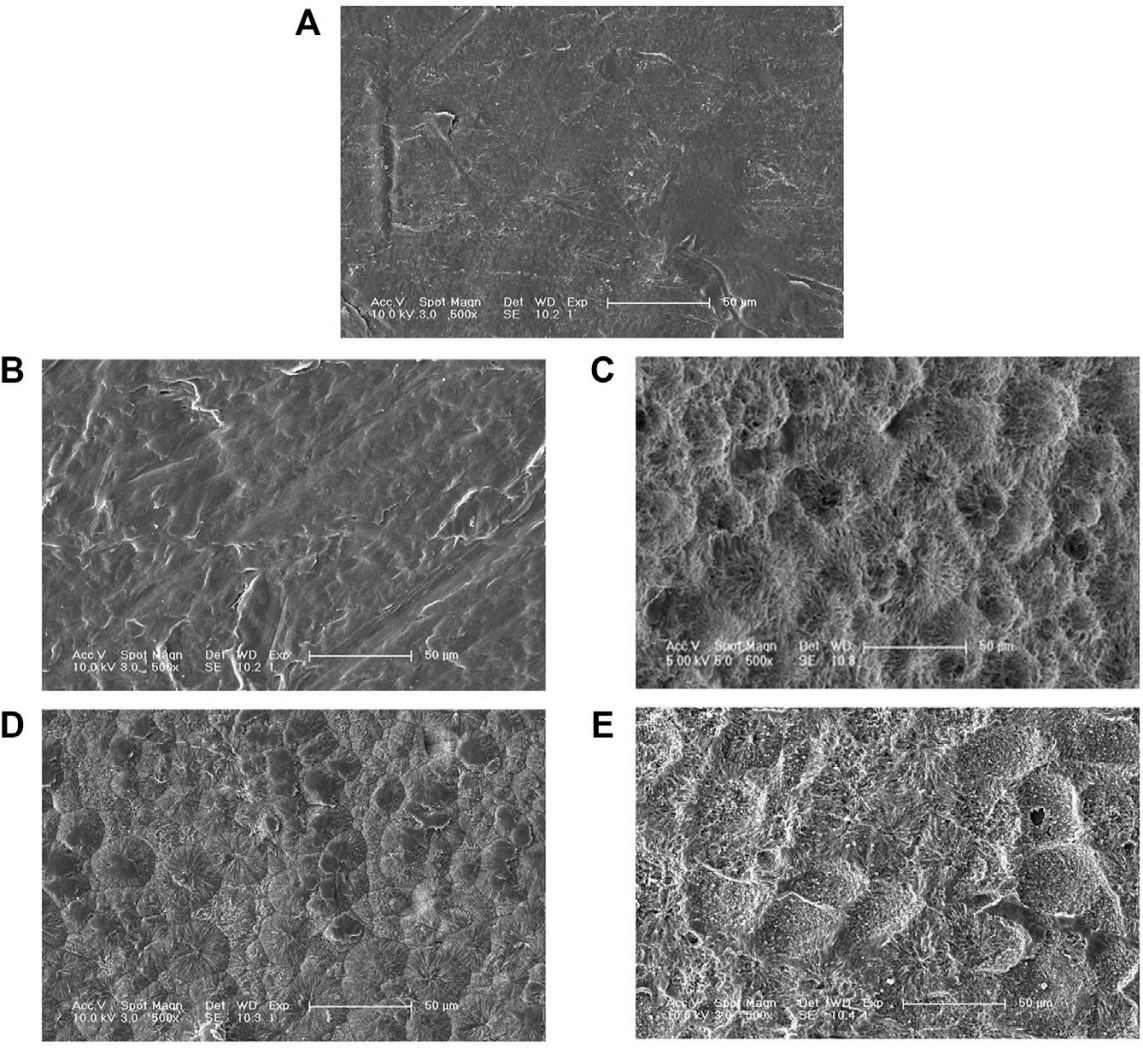
SEM micrographs of undegraded PCL **(A)**, degraded by PCLase I [**(B)** 36 h; **(C)** 72 h], and PCLase II [**(D)** 5 days; **(E)** 8 days].

SEM micrographs of the PCL films degraded by the two purified enzymes are shown in Figure 5. The PCL film’s surface was smooth before enzymatic hydrolysis. However, as the enzymatic hydrolysis time increased; the film surface was damaged to varying degrees. The appearance of spherulites in the degradation process showed that the PCL-degrading enzyme preferentially degrades the film’s amorphous regions, and then degrades the crystalline regions. The SEM results also showed that aside from the degradation rates of PCLase I and PCLase II, their degradation modes also vary. Figure 5B and 5D show that when the degradation rate was 30%, the film degraded by PCLase I appeared to be a lamellar structure with no spherulites, whereas the film degraded by PCLase II presented spherulites, suggesting that the action of PCLase II may be deeper.

### Cloning, Expression, and Sequence Analysis of PCL-Degrading Enzymes

The two purified PCL-degrading enzymes were analyzed by MALDI-TOF-MS. The peptide fingerprints showed that PCLase I had 100% similarity to a hypothetical protein (WP_004373894.1) from *Pseudomonas mendocina* ymp and that PCLase II had 100% similarity to a lactonizing lipase (WP_003239806.1) from the same strain. The primers were designed according to the homologous sequence and applied for PCR amplification using the genome of *P. hydrolytica* sp. DSWY01^T^ as template. The sequencing results showed that the amplified fragment sequences were consistent with the homologous sequence derived from *P. mendocina* ymp. Amplified genes, excluding the putative signal peptide sequence, were cloned and expressed in *E. coli* (DE3). The detection showed that the expressed recombinant enzymes have PCL-degrading activity, and their molecular weights were consistent with the previously purified PCLase I and PCLase II. This result indicated that the two DNA fragments (i.e., *pcl1* and *pcl2*) obtained through peptide fingerprinting and homologous amplification were indeed genes encoding PCLase I and PCLase II.

*Pcl1* comprised an ORF of 843 nucleotides encoded with a protein of 280 residues, with a theoretical molecular weight of 29.63 kDa and a theoretical pI of 8.55, predicted with a 22-amino acid signal peptide at the N-terminal region. *Pcl2* comprised an ORF of 954 nucleotides encoded with a protein of 317 residues, with a theoretical molecular weight of 33.20 kDa and a theoretical pI of 6.59, predicted with a 24-amino acid signal peptide at the N-terminal region. Both enzymes belonged to *α*/*β* hydrolase family and contained catalytic triad composed of Ser148-Asp198-His228 and Ser111-Asp261-His283 in the active center, respectively. The two enzymes both had a single catalytic structure with no substrate binding domain, which is different from solid-phase substrate hydrolases, such as cellulase and polyhydroxybutyrate depolymerase (Mattinen et al., 1997; Shin et al., 2002; Hiraishi et al., 2006).

## Discussion

*P. hydrolytica* sp. DSWY01^T^ is a strain screened in our laboratory in a previous research. This strain has been found to have the ability to degrade polyesters, including PHB and PLA (Zhou et al., 2020). In the current study, two PCL-degrading enzymes were purified from the strain’s fermentation supernatant. These results indicated that the strain had good application potential in the degradation, recycling, and transformation of polymer polyesters.

**FIGURE 6:**
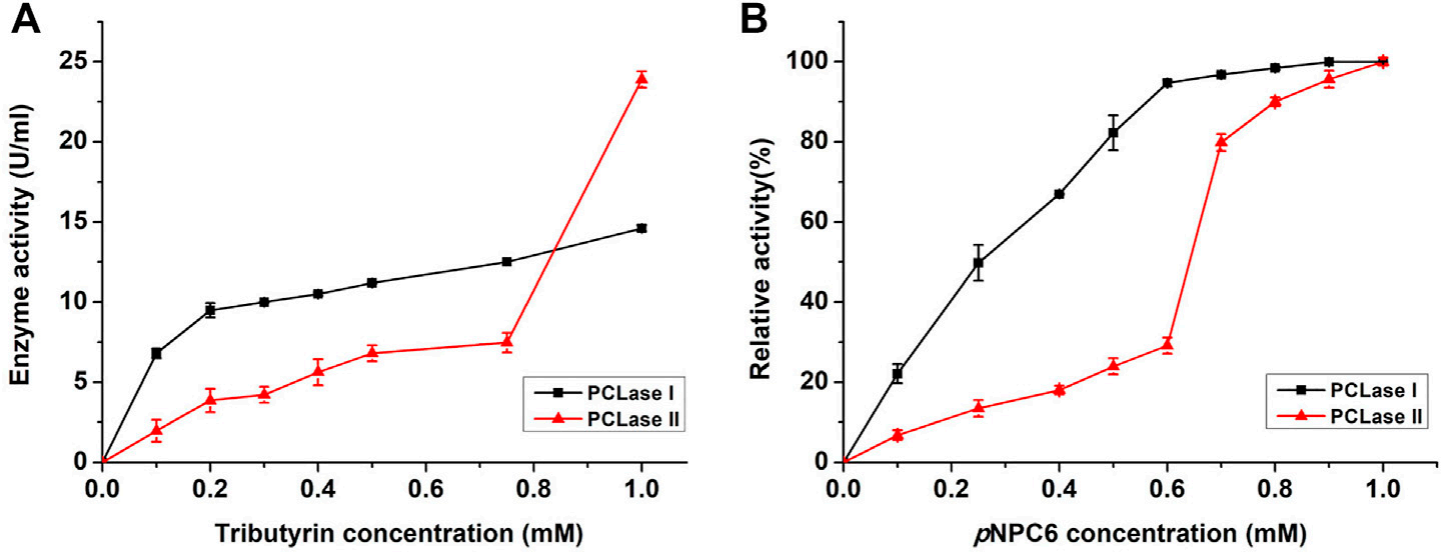
Effects of tributyrin and *p*NP ester on PCL-degrading enzymes. Tributyrin **(A)** and *p*NP esters **(B)** were used as substrates to detect the activity of PCLase I and PCLase II under different concentrations.

Microorganisms usually secrete isoenzymes to synergistically degrade macro-molecules *in vitro*. However, considering that PCL is a chemically synthesized polyester, the original function of the PCL-degrading enzymes secreted by microorganisms should not be to degrade PCL. Several enzymes secreted by the strain that degrade natural polyester more likely happened to have relatively broad substrate specificity, which recognized the PCL substrate and further broke the ester bond. The substrate specificity analysis results showed that both PCL-degrading enzymes degraded ester substrates, including olive oil and tributyrin; PCLase I significantly degraded the rough cutin of apple peel, whereas PCLase II had no such degradation ability. In addition, sequence alignment showed that PCLase II may be a lipase and that PCLase I was most similar to a hypothetical protein with an unidentified function. Considering these characteristics of the enzymes, we speculated that these two PCL-degrading enzymes possibly belong to cutinase and lipase families, respectively. To further study the properties of the PCL-degrading enzymes, we used tributyrin as substrate in an interface activity experiment. As shown in Figure 6, the degradation activity of PCLase I increased with the increase of the substrate concentration and showed no interface activation effect; however, the activity of PCLase II increased sharply when the tributyrin concentration was higher than 0.8 mM, showing a typical interface activation effect. This results support earlier suggestion that PCLase II is a lipase whereas PCLase I is a cutinase.

**FIGURE 7:**
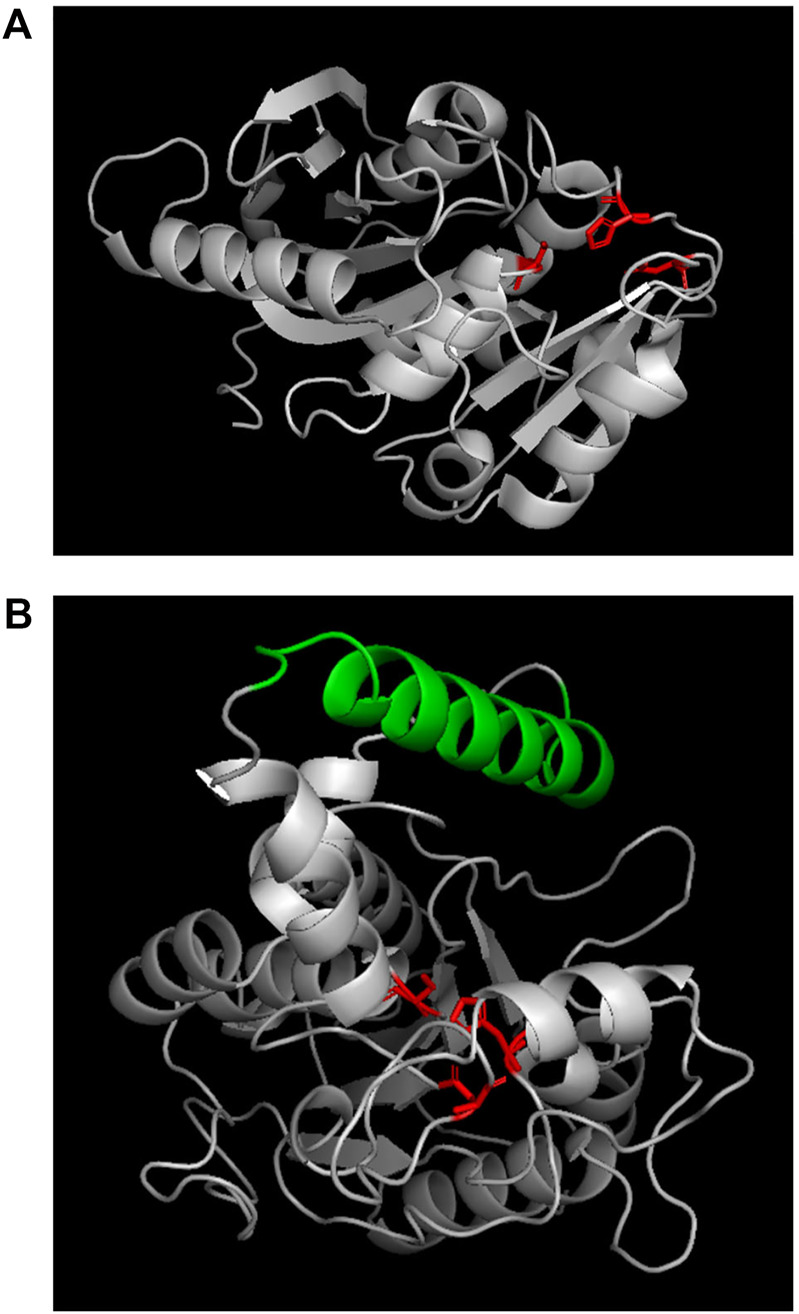
Structures of PCLase I **(A)** and modeled PCLase II **(B)**. Catalytic triad is marked in red and “lid structure” is colored in green.

Previous research found that lipases usually have catalytic triad composed of nucleophilic-histidine acid residues; and in its open conformation, the “lid structure” at the top of the active site enables the solvent to approach the catalytic cavity centre (Brzozowski et al., 1991; Grochulski et al., 1994; Brzozowski et al., 2000). By contrast, the active region of cutinase is directly exposed to the top of the three-dimensional structure and can directly contact and interact with the substrate. In the present study, the enzymes’ sequences were blasted in the database, and the results showed that PCLase I is an enzyme with a known structure (PDB No. 2fx5) (Sibille et al., 2006) (Figure 7A) and that although PCLase II has no structural information, PCLase II is similar to the lipase PAL (PDB No. 1ex9.1) derived from *Pseudomonas aeruginosa* PAO1 with the sequence identity of 82.75%. We modeled the structure of PCLase II and then compared the differences in the structure of the two PCL-degrading enzymes interacting with the substrate.

Lipase PAL (lex9.1.A) was used as a template for homologous modeling in SWISS-MODEL. The sequence alignment results between PAL and PCLase II showed that Amino Acids 30 to 317 of PCLase II corresponded to amino acids 1 to 285 of the template PAL. Unlike the *α*-helix in PAL, Amino Acids 226 to 232 of PCLase II formed a *β*-sheet. However, other components corresponded well to the template. A previous study revealed that PAL has a movable helical structure formed by amino acids 125–147 (Nardini et al., 2000). This structure and the surrounding structure are the key “lid structures” that can be used to control the exposure of the enzyme active site, which makes the solvent and the substrate enter the cavity where the active site is located. The helical structure of PCLase II’s 155–180 amino acids is highly similar to PAL’s “lid structure” (Figure 7B). Combined with PCLase II’s properties, we speculate that PCLase II belongs to lipase and that its “lid structure” may be related to the “interface activation” effect. In addition, this lid structure may also play a steric hindrance, preventing the long-chain polyester from entering the active pocket of the enzyme. This limitation may be the reason why the degradation efficiency of PCLase II to PCL film was lower than that of PCLase I.

The MS analysis results showed the presence of dimers, trimers, and tetramers in the enzymatic hydrolysis products, suggesting that these enzymes can bind to long-chain polyesters. Moreover, these enzymes can also degrade PBS polyester, indicating that the enzymes have excellent substrate versatility, which makes these enzymes have the potential to be used in the degradation and recycling of mixed biodegradable polyesters. Moreover, these properties of enzymes are valuable to the development and synthesis of derivatized polyesters based on PCL or PBS structures. In addition, these properties of enzymes make the degradation of derivatized polyesters based on PCL or PBS structures possible, which has guiding significance for the development of more types of biodegradable polyesters.

## Data Availability

The original contributions presented in the study are included in the article/Supplementary Material, further inquiries can be directed to the corresponding author.
